# Identification of transcriptomics biomarkers for the early prediction of the prognosis of septic shock from pneumopathies

**DOI:** 10.1186/s12879-021-06888-w

**Published:** 2021-11-26

**Authors:** Songchang Shi, Xiaobin Pan, Hangwei Feng, Shujuan Zhang, Songjing Shi, Wei Lin

**Affiliations:** 1 Department of Critical Care Medicine, Shengli Clinical Medical College of Fujian Medical University, Fujian Provincial Hospital South Branch, Fujian Provincial Hospital Jinshan Branch, Fuzhou, 350001 Fujian People’s Republic of China; 2 Department of Endocrinology, Shengli Clinical Medical College of Fujian Medical University, Fujian Provincial Hospital, No.134 East Street, Gulou District, Fuzhou, 350001 Fujian People’s Republic of China; 3 Department of Critical Care Medicine, Shengli Clinical Medical College of Fujian Medical University, Fujian Provincial Hospital, No.134 East Street, Gulou District, Fuzhou, 350001 Fujian People’s Republic of China

**Keywords:** Biomarkers, Transcriptomics, Prediction, Prognosis, Septic shock

## Abstract

**Background:**

Identifying the biological subclasses of septic shock might provide specific targeted therapies for the treatment and prognosis of septic shock. It might be possible to find biological markers for the early prediction of septic shock prognosis.

**Methods:**

The data were obtained from the Gene Expression Omnibus databases (GEO) in NCBI. GO enrichment and KEGG pathway analyses were performed to investigate the functional annotation of up- and downregulated DEGs. ROC curves were drawn, and their areas under the curves (AUCs) were determined to evaluate the predictive value of the key genes.

**Results:**

117 DEGs were obtained, including 36 up- and 81 downregulated DEGs. The AUC for the *MME* gene was 0.879, as a key gene with the most obvious upregulation in septic shock. The AUC for the *THBS1* gene was 0.889, as a key downregulated gene with the most obvious downregulation in septic shock.

**Conclusions:**

The upregulation of *MME* via the renin-angiotensin system pathway and the downregulation of *THBS1* through the PI3K–Akt signaling pathway might have implications for the early prediction of prognosis of septic shock in patients with pneumopathies.

## Background

Septic shock, characterized by circulatory and cellular abnormalities, is associated with substantial morbidity and mortality [1]. The worldwide mortality rate of septic shock was > 40% in 2016 [2, 3], making septic shock a major healthcare problem globally. It plays an important role in the morbidity and mortality of patients in the intensive care unit and results in substantial health care costs [4, 5].

Although much progress has been made in diagnosing and treating septic shock, mortality remains unacceptably high [6]. A consistent increase in sepsis has been shown over the past two decades [7], its incidence is predicted to increase rapidly because of the sepsis observed in COVID-19 [8]. It is known that certain genes and signaling pathways participate in the occurrence of septic shock in children [9]. Identifying biological subclasses of septic shock might provide specific targeted therapies for the treatment and prognosis of septic shock [10, 11]. Studying the molecular mechanism of septic shock is certainly important [9].

We hypothesized that key genes are involved in the early stage of septic shock. These genes might be involved in the initiation of septic shock, and the mediated molecular changes might affect the prognosis of the disease. Therefore, it could be possible to detect the changes in gene expression in the early stage of septic shock through gene chips. By correlating these changes with the prognosis of the disease, it might be possible to identify biological markers for the early prediction of the prognosis of septic shock.

## Materials and methods

### Data collection

**Fig. 1:**
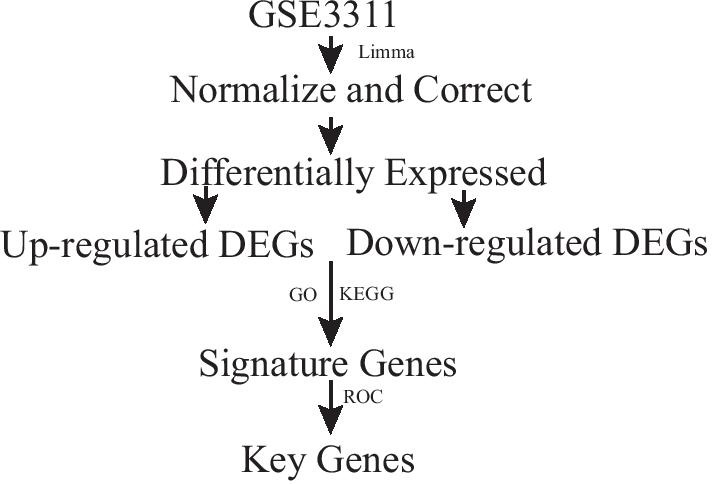
Study flowchart

The data was obtained from the Gene Expression Omnibus databases (GEO) in NCBI, a public functional genomics data repository. The expression dataset GSE33118 (https://www.ncbi.nlm.nih.gov/geo/; GSE33118) was obtained from the Affymetrix GPL570 platform (Affymetrix Human Genome U133 Plus 2.0 Array), which was submitted by Raffelsberger et al. Septic shock by pneumopathy was studied prospectively in 20 patients, whose blood samples were taken within 12 h of diagnosis. Preparation, processing, and analysis of data were performed using the R software (version 3.6.3). The flowchart of this study is shown in Fig. 1.

### Identification of differentially expressed genes

The limma package in R was used to normalize and correct the data [12]. The expression data were screened for differentially expressed genes (DEGs) between different outcomes with the criteria of |log2fold change (FC)|> 1 and adjusted P-value < 0.05 [13]. Then, the identified DEGs were divided into upregulated and downregulated DEGs.

### DEGs analyze via GO and KEGG

Gene Ontology (GO) [14] enrichment and Kyoto Encyclopedia of Genes and Genomes (KEGG) [15–17] pathway analysis were performed to investigate the functional annotation of the up- and downregulated DEGs. The expression matrix of the up- and downregulated DEGs were analyzed by GO and KEGG enrichment to determine whether they show statistically significant differences between different outcomes. A P-value < 0.05 was considered statistically significant.

### ROC analysis of the significantly enriched DEGs

Up- and downregulated DEGs were considered candidate genes to predict the outcome. Receiver operating characteristic (ROC) curves were drawn. Their areas under the curve (AUCs) were determined to evaluate the predicted value of the key genes using the pROC package in R [18].

## Results

### Identification of DEGs

**Fig. 2:**
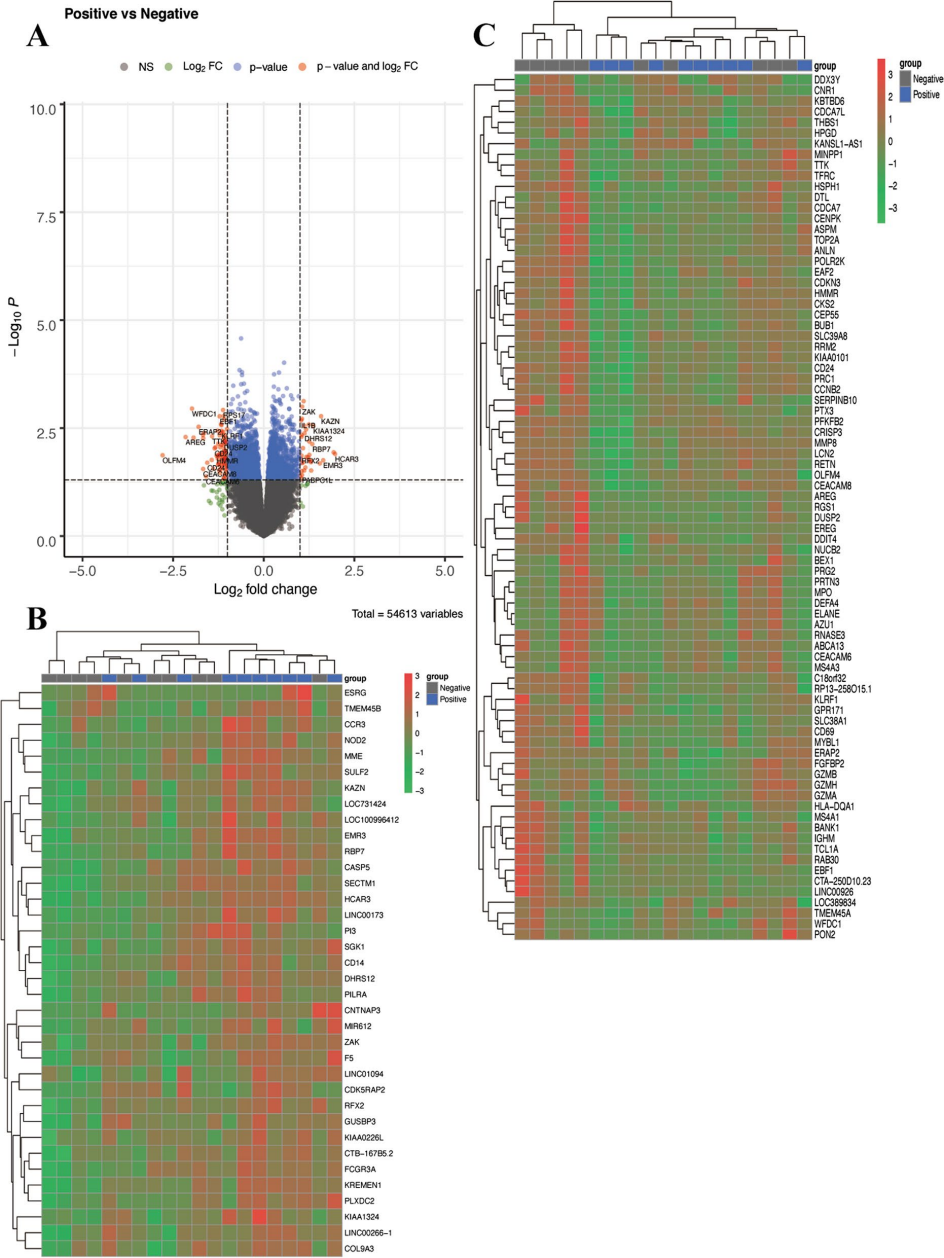
Differentially expressed genes. **A** Genes differentially expressed between outcome. **B** The first 36 of up-regulated DEGs. **C** The first 81 of down-regulated DEGs

There were 54,613 genes in the GSE33118 dataset, including 29,647 upregulated and 24,939 downregulated genes. Finally, 117 DEGs were identified, including 36 up- and 81 downregulated DEGs. The volcano map of all DEGs is displayed in Fig. 2A. The heatmaps of the top 36 upregulated genes and the top 81 downregulated genes are displayed in Fig. 2B, C.

### GO enrichment and KEGG pathway of DEGs

**Fig. 3:**
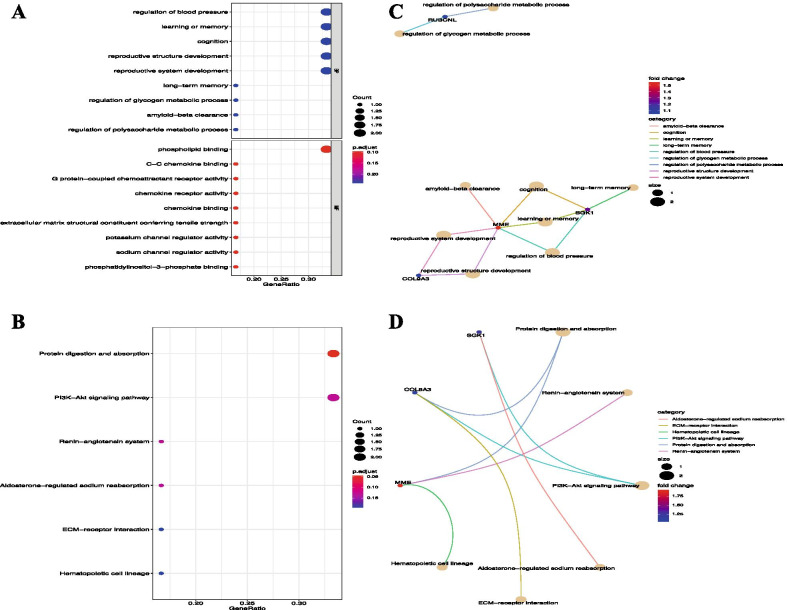
Enrichment function of upregulated DEGs via GO and KEGG. **A**, **B** Enrichment of GO. **C**, **D** Enrichment of KEGG

GO categories and KEGG pathways were used to evaluate the up- and downregulated genes. The upregulated DEGs were significantly enriched in Molecular Function (MF), such as phosphatidylinositol-3-phosphate binding, sodium channel regulator activity, potassium channel regulator activity, extracellular matrix structural constituent conferring tensile strength, chemokine binding, chemokine receptor activity, G protein-coupled chemoattractant receptor activity, and C–C chemokine binding, as well as in Biological Process (BP), such as regulation of polysaccharide metabolic process, amyloid-beta clearance, regulation of glycogen metabolic process, and long-term memory (Fig. 3A). The KEGG pathway results revealed that the upregulated DEGs were significantly enriched in the renin-angiotensin system, aldosterone-regulated sodium reabsorption, ECM-receptor interaction, and hematopoietic cell lineage (Fig. 3B). Among those results, we will focus on the key genes, such as *MME*, *SGK1*, and *COL9A3* (Fig. 3C, D).

**Fig. 4:**
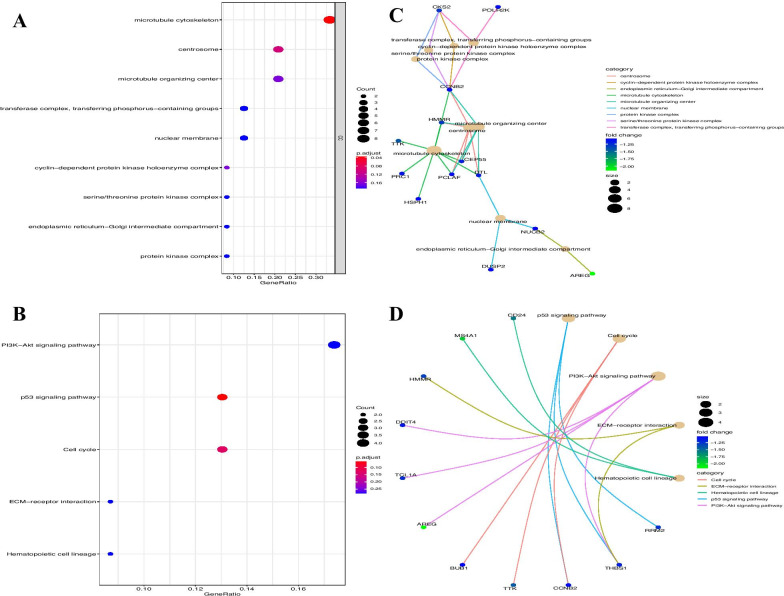
Enrichment function of downregulated DEGs via GO and KEGG. **A**, **B** Enrichment of GO. **C**, **D** Enrichment of KEGG pathway

The downregulated DEGs were significantly enriched in cellular components (CC), such as protein kinase complex, endoplasmic reticulum-Golgi intermediate compartment, serine/threonine-protein kinase complex, and cyclin-dependent protein kinase holoenzyme complex (Fig. 4A). The KEGG pathway results showed that the downregulated DEGs were significantly enriched in the PI3K–Akt signaling pathway, p53 signaling pathway, cell cycle, ECM-receptor interaction, and hematopoietic cell lineage (Fig. 4B). We will focus on the key genes, such as *CD24*, *MS4A1*, *HMMR*, *DDIT4*, *TCL1A*, *AREG*, *BUB1*, *TTK*, *CCNB2*, *THBS1*, and *RRM2* (Fig. 4C, D).

### Identification of the key genes associated with the outcome

**Fig. 5:**
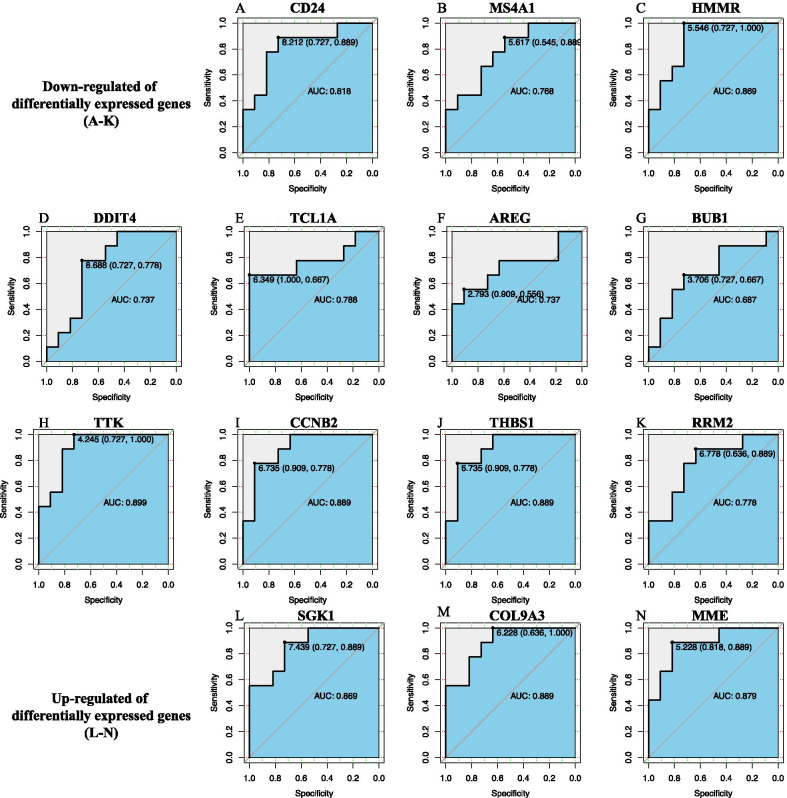
ROC of key genes

There were three upregulated and 11 downregulated key genes. Among them, all the key genes showed that they could predict the outcome accurately with AUCs > 0.7, except for *BUB1* (Fig. 5). The AUC for the *MME* gene was 0.879, as a key gene with the most obvious change in expression among the upregulated genes. The AUC for the *THBS1* gene was 0.889, as a key downregulated gene with the most obvious change in expression among the downregulated genes. The prognostic cutoff concentration to predict the outcome was chosen after ROC analysis (Fig. 5).

## Discussion

Sepsis is a main cause of critical illness and mortality all over the world [19, 20]. Septic shock reflects a more severe illness with a higher likelihood of death than sepsis alone [1]. Septic shock results in multiorgan dysfunction, including the liver, kidney, and lung [1]. Clinical symptoms of early septic shock are often nonspecific, so they are easy to miss. The identification of the early signs of septic shock might help a timely diagnosis and initiate therapy faster [21, 22]. This study examined the biological subclasses using bioinformatics and could provide a foundation for the molecular diagnosis and prognosis of septic shock from pneumopathies.

In this study, as a significantly upregulated gene, *MME* (membrane metalloendopeptidase) might play an important role in the prognosis of septic shock through the renin-angiotensin system pathway [23], which is activated to increase the arterial blood pressure in sepsis [24]. Indeed, MME can produce Ang-(1–7) (an active form of angiotensin) from Ang-(1–9) [23]. Ang-(1–7) is a potent vasodilator that might be participating in the dramatic drop in blood pressure often observed in septic shock [25]. Still, Ang-(1–7) appears to have protective actions in sepsis [26]. On the other hand, MME has also been involved in Ang-(1–12) metabolism, which is known to activate the renin-angiotensin system [23]. Excessive activation of the renin-angiotensin system might further worsen the outcomes of sepsis [27]. Fernandes et al. revealed that a partial blockade of the renin-angiotensin system might provide an opportunity to improve the outcomes of sepsis-induced refractoriness to vasoconstrictors [28]. Furthermore, the renin-angiotensin system has been recognized to play a major role in some biological processes such as coagulation, apoptosis, and inflammation [29, 30]. Still, the exact role of MME in sepsis remains to be determined.

In this study, as a significantly downregulated gene, *THBS1* might play an important part in the prognosis of septic shock through the PI3K–Akt signaling pathway. Mizuta et al. [31] suggested that activating the PI3K/Akt signaling pathway contributes to suppressing endothelial apoptosis, inhibits lung hemorrhage and edema, and improves murine survival. The morbidity and mortality caused by myocardial infarction might be decreased via the stimulation of the PI3K/Akt-dependent cascade [32]. It was revealed that suppressing inflammatory and antioxidant responses through the PI3K/Akt pathway might mitigate the effects of sepsis [33]. In this study, besides *THBS1*, *DDIT4*, *TCL1A*, and *AREG* might be biological markers in the prognosis of septic shock by acting via PI3K/Akt signaling pathway. Still, the role of THBS1 in the regulation of the PI3K–Akt signaling pathway in cancer is relatively well defined [34], but its role in sepsis remains to be determined.

Different metabolomic patterns might have a major impact on the development of novel diagnostic methods for the early diagnosis and prognosis of septic shock [35]. The heterogeneity between DEGs and the specific signaling pathways prompted us to select one gene to be used as a prognostic marker of the outcome. In this study, prognostic cutoff concentrations for predicting the outcome were selected using a ROC curve analysis.

The potential limitations of our study should be considered. The datasets we used were obtained from a public database. This dataset only included data about septic shock from pneumopathies. All results were obtained using bioinformatics. Future studies are currently being designed for examining the exact roles of MME and THBS1 in the renin-angiotensin system and PI3K/Akt signaling pathways in septic shock from pneumopathies. Further experiments are needed to verify the results of this study and expand the generalizability to septic shock from other sources.

## Conclusion

In summary, the upregulation of *MME* and its role in the renin-angiotensin system pathway and the downregulation of *THBS1* and its role in the PI3K–Akt signaling pathway might have important implications for the early diagnosis and prognosis of septic shock from pneumopathies. This study suggests that the molecular typing of septic shock could reveal diagnostic, prognostic, and therapeutic biomarkers for patients with septic shock, allowing early diagnosis and management. Still, the biomarkers need to be refined, and prognosis models need to be built.

## Data Availability

The datasets analyzed during the current study are publicly available in the [Gene Expression Omnibus databased] repository/database, [https://www.ncbi.nlm.nih.gov/geo/; GSE33118].
